# Comparison of the effectiveness and efficiency of the grid and link search methods to recover scattered skeletal remains

**DOI:** 10.1007/s00414-024-03247-7

**Published:** 2024-05-10

**Authors:** Craig Adam Keyes

**Affiliations:** Department of Forensic Medicine and Pathology, School of Clinical Medicine, Faculty of Health Sciences, University of the Witwatersrand, Johannesburg, South Africa

**Keywords:** Forensic anthropology, Forensic taphonomy, Search methods, Scavenging, Bone dispersal

## Abstract

The grid and link search methods are used to recover scattered skeletal remains. Neither have not been compared robustly and clear guidelines for the link method have not been sufficiently developed. The study aimed to compare the effectiveness and efficiency of both methods and propose guidelines for the link method. The scattering patterns of two scavengers of forensic relevance—slender mongooses (*Galerella sanguinea)* and black-backed jackals (*Canis mesomelas*)—were recreated using four pig skeletons (*Sus scrofa domesticus*). Two groups (*n* = 6 each) were assigned a different method to recover the scattered remains. The length of the search and when each bone was located for each scatter pattern was recorded for each group and scatter pattern. A Likert scale questionnaire assessed participants’ perceptions of their assigned method. A paired t-test (*p* = 0.005) compared the efficiency of each method and the questionnaire answers. Both methods were effective, recovering 100% of all remains. The link method was more efficient for both scatter patterns, despite there being no statistical significance (jackal: *p* = 0.089; mongoose: *p* = 0.464). Participants indicated favorable views for both methods; however, the link method scored significantly more favorably (*p* = 0.01) for efficiency. Specific guidelines were developed for the use of the link method. The link method is suggested for the recovery of scattered remains in forensic contexts, especially when the scavenger, its behavior, and scattering pattern is known or suspected.

## Introduction

A critical part of any medico-legal death investigation is the recovery of the postmortem human remains. The quality of a scene’s management will determine the quality and quantity of evidence recovered for forensic analysis. This can be impacted by animal interference. Despite global urbanization and conservation efforts, proximity and interaction between humans and animals continues [1, 2]. Forensic investigations often encounter evidence of animal scavenging of human remains before they can be recovered for analysis. Such remains are often discovered by pedestrians, excavations, and domestic dogs [3]. The prevalence of such cases was highlighted in a United Kingdom survey of police specialists [4], which indicated that 53% of participants had previously been involved in the search for scavenged and scattered human remains [4]. A large proportion (80.83%) of the police specialists also indicated that they were unable to recover all of the human skeletal remains, even when they were assisted by a cadaver dog [4].

Scattering of remains by scavengers may limit the number of skeletal remains recovered. This substantially impacts the effectiveness of a forensic investigation. Unfortunately, there are no international standardized protocols for the search and recovery of scattered human remains in forensic investigations. A common search method employed in the search for surface remains in an outdoor environment is the walk-the-line method in a grid pattern [3], which is used in anthropology and archeology. This method is also referred to as a visual survey, pedestrian survey, or walkover [3]. For this article, it will be referred to as the grid search method. This method involves several individuals, standing equal distance apart from each other (1 m is suggested) [3], as they walk in a straight line - flagging and recording scattered remains in situ as they are recovered [5]. Once the team reaches the end of the designated strip of area, they reverse their direction and search again in an adjacent parallel strip [3]. Once the full length of the designated area has been searched, this process is repeated over the same area at 90 degrees to the previous search line. This increases the chances of remains being discovered because the area is searched twice from two different angles [3]. Walking the line in just one direction has proven to be 84% effective in the recovery of remains [3]. Changing the direction and searching the same area again (grid method) increases the recovery rate to 90% [3]. Despite this method being effective, it is very rigid in its application and can be very time-consuming when remains are scattered over a large area. Other factors that impact the success rate of the grid method includes the type and size of the object being searched for, the speed of the searchers, the position of the sun in relation to the searchers (walking towards or away from the sun) [6], the terrain, and cumulative fatigue of the searchers [7]. Smaller skeletal remains can be difficult to recover, especially when ground cover by leaf litter and other dense vegetation may obscure these smaller elements. This challenge remains even when using wire mesh for the screening of such vegetation and their decomposition deposits [7]. Such plant litter can also stain the skeletal remains making it difficult to distinguish between bone, vegetation, and understory [3].

The time afforded to forensic investigations can be limited by several variables such as available resources, weather, and available personnel. In such cases, there is a need for a search method that is more time efficient. It has been suggested that if an animal’s scattering pattern is consistent and known, a more effective and time efficient search method could potentially be devised [8]. This is supported by a study by Young et al. [9], who discovered that investigating officers are twice as successful in the recovery of skeletal remains if they have an understanding of scavenging behaviors than those without the same knowledge [9]. A link search method has been suggested as an alternative to the grid search method for the recovery of remains that have been scattered by animals [8]. This method is far more flexible than the grid search method, as it relies on the searcher(s) to adjust their search direction as prompted by cues created by the scavengers, such as drag marks in the soil, clumps of fur, animal scat, and game trails [8]. Searchers follow the most likely path of the scavenger by following the scattered remains and scavenger-created evidence. As a skeletal element is located, the searcher adjusts the direction of their movement depending on visual cues or observations that suggest where the next skeletal element may be [10]. When using this method it is recommended that searchers have a basic knowledge of scavenging behaviors, which will increase the chances of searchers identifying scavenger clues [9].

Unfortunately, previous publications have not provided detailed guidelines for the link search method, including how to perform the method and its setup, and no studies have sufficiently assessed its effectiveness. The aim of this study was to compare the effectiveness and efficiency of the grid and link search methods in recovering scattered skeletal remains, and to propose guidelines on how to perform the link search method.

## Methods

The sample included the skeletons of four pig carcasses (*Sus scrofa domesticus*) that had been recovered after they had been scavenged and scattered in two previous studies [11, 12]. Two skeletons were scavenged by slender mongooses (*Galerella sanguinea)* [12] and two were scavenged by black-backed jackals (*Canis mesomelas*) [11]. The scattering patterns of each scavenger were mapped prior to the recovery of their scavenged pig carcasses in the initial studies [11, 12].

**Fig. 1:**
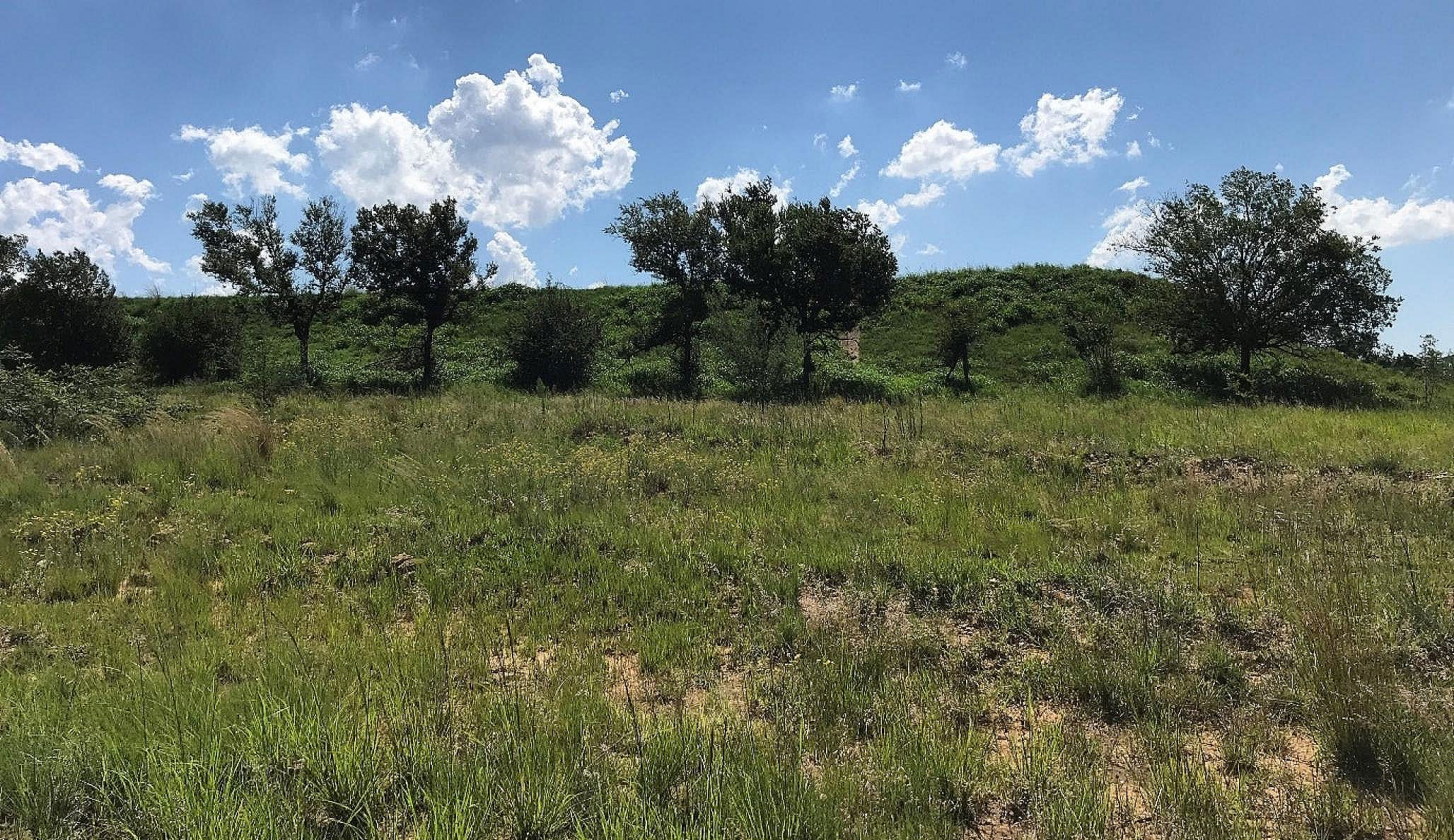
Frankenwald research site

The present study was performed at the Frakenwald Research Site (Fig. 1), which is a research site for forensic taphonomic research in Johannesburg, South Africa [12, 13]. This environment replicates those where most scattered human remains are recovered from in a South African forensic context (open grassland with low shrubbery and sparce trees).

**Fig. 2:**
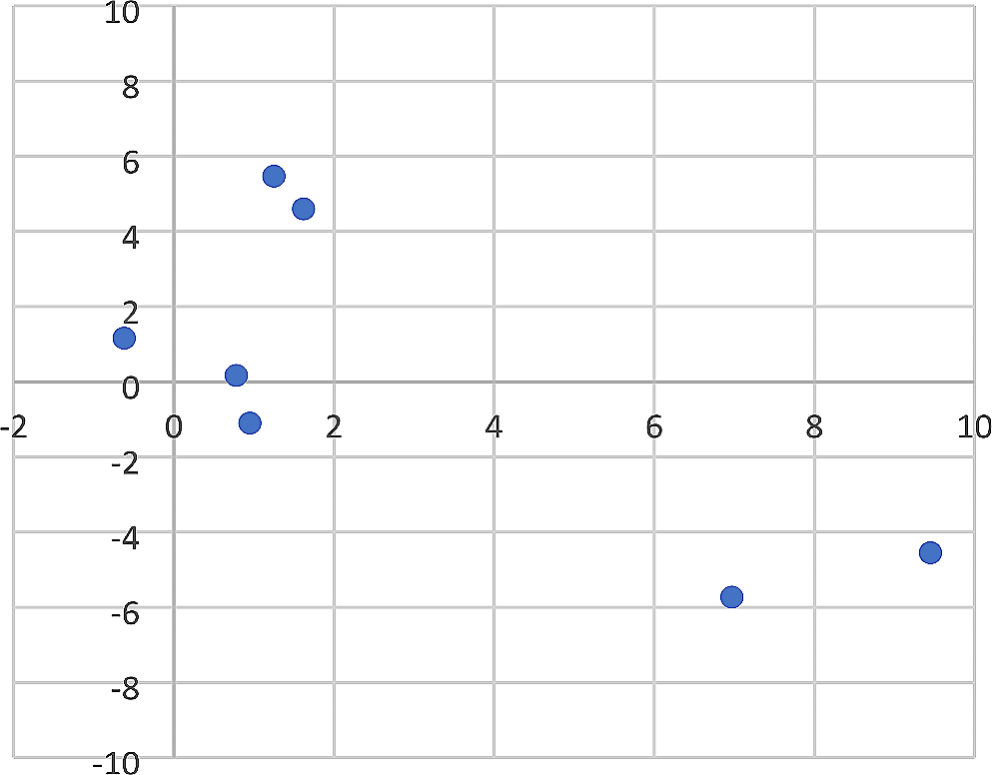
Slender mongoose scattering pattern on a north-south y-axis and east-west x-axis. (Points indicate individual skeletal elements or a grouping of skeletal elements in very close proximity or an articulation of skeletal elements. Origin 0.0 indicates the original site of deposition prior to scattering. Distance is measured in meters)

**Fig. 3:**
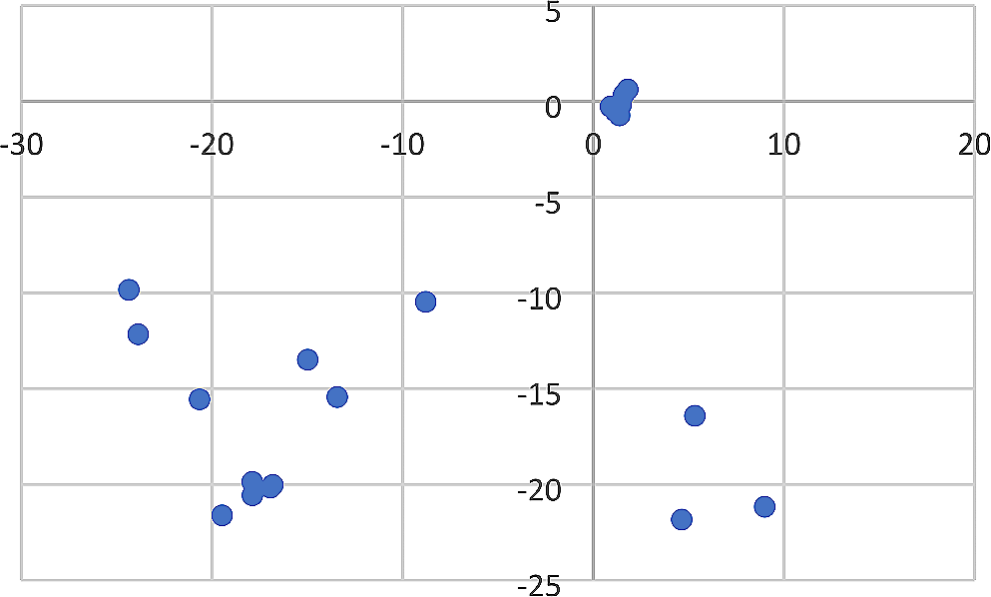
Black-backed jackal scattering pattern on a north-south y-axis and east-west x-axis. (Points indicate individual skeletal elements or a grouping of skeletal elements in very close proximity or an articulation of skeletal elements. Origin 0.0 indicates the original site of deposition prior to scattering. Distance is measured in meters)

The scattering pattern of each scavenger was recreated in duplicate (one for the grid search and one for the link search) in a separate location at the research site. The slender mongoose scattering included 17 skeletal elements scattered over an area of 113m^2^ (Fig. 2). The black-backed jackal scattering included 28 skeletal elements scattered over an area of 701m^2^ (Fig. 3). Game trails created by the scavenging animals in the initial studies [11, 12] were recreated to ensure that visual cues observed in the previous studies were present in the present study. Apart from recreating game trails, the impact of vegetation was not specifically accounted for in this study because the environment was a replicate of the initial studies [11, 12], which included areas with long dry grass.

The study participants included twelve Bachelor of Health Sciences Honours in Forensic Sciences students at the University of Witwatersrand. The participants were randomly divided into two groups, each of which was assigned a different search method to recover their assigned pair of slender mongoose and black-backed jackal scattered remains. The first group performed the grid search method, and the second group performed the link search method. Each group was trained separately on how to perform their respective search method.

The grid search method does not require any context or information regarding the environment, the scavenging animals, or their behaviors. As such, the group assigned this method was not provided with any context and was instructed to follow the grid search method.

The link search method requires knowledge of possible scavenging animals present in the environment and their scattering behaviors. The group assigned this method was informed that the common scavenger guild that inhabits urban and peri-urban South African veld (i.e., grassland) environments included common large-spotted genet (*Genetta tigrine*), black-backed jackal (*Canis mesomelas*), slender mongoose (*Galerella sanguinea*), water mongoose (*Atilax paludinosusis*), yellow mongoose (*Cynictis penicillata*), Cape porcupine (Hystrix africaeaustralis), honey badger (*Mellivora capensis*), and domestic dogs (*Canis familiaris*). The group was informed of the general scattering behaviors of dominant mammalian scavengers common in South African veld environments. This included the scattering of remains in two different but uniform directions, with the two directions forming a 90-de-gree arc the original deposition site (or site where a body or carcass was originally whole, prior to dismemberment or disarticulation by scavengers) [11, 12]. They were also informed that scavengers often scatter remains along game trails towards a burrow or beneath foliage [11, 12]. The group was also informed that the scattering range was relative to the size of the dominant scavenging species [11, 12].

Each group was given a data collection sheet to record when and which skeletal element was located and flags to mark the location of the located skeletal element. Participants were not informed on how many bones were present at the site, nor were they given a time limit to search for the remains. Each group was asked to record what time they started the search, when each bone was located, and when they ended their search. Each group was first transported to their assigned site with remains scattered in the recreated black-backed jackal scattering pattern. This was followed by their transportation to their respective site with remains scattered in the recreated slender mongoose scattering pattern.

At the completion of the recovery at both sites, participants completed a questionnaire that reviewed their experiences with their assigned search method. The questionnaire was comprised of eight questions in the form of a five-point Likert scale. The questionnaire assessed the participants’ perception of the effectiveness of each search method. Each participant signed a participation consent form that informed them that there were no personal risks or benefits to participating in the study, that no personal information was recorded, all answers were anonymous, they were under no obligation to participate, and they could withdraw from the study at any stage.

Descriptive statistics were used to determine the effectiveness of each search method by comparing the number of skeletal elements collected and a paired t-test (5% level of significance) was used to compare the time of recovery of each skeletal element and the Likert scale answers for each question in the questionnaire.

Ethical clearance for the study was granted by the University of the Witwatersrand’s Animal Research Ethics Committee (waiver numbers: 17-04-2018-O and 2021-04-04-O).

## Results

### Effectiveness and efficiency and of the grid and link search methods

The grid search method and the link search method were effective in the recovery of all scattered skeletal elements for both scattering patterns (100% respectively).

**Fig. 4:**
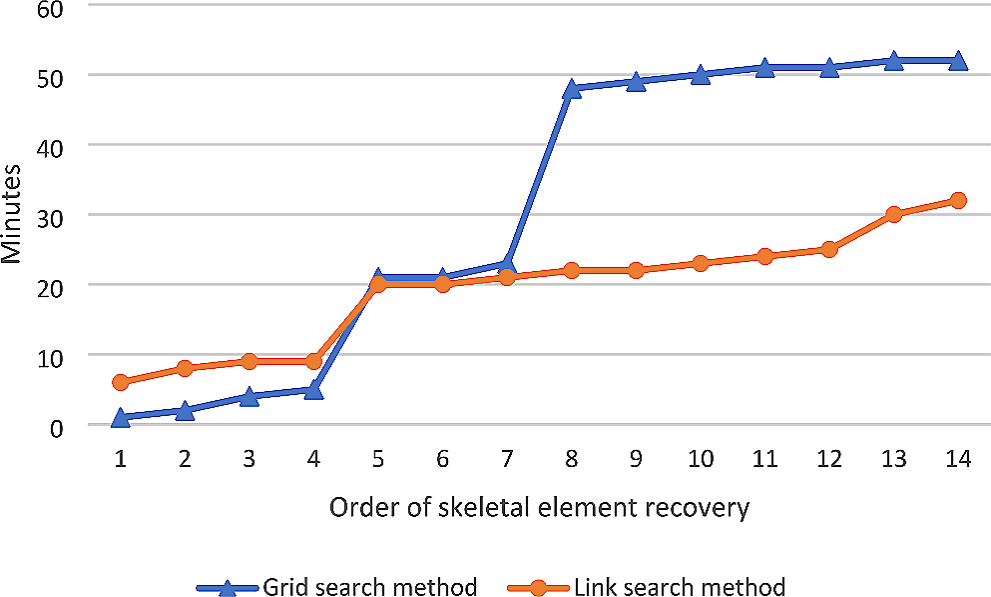
Comparison of the length of time (min) to recover each scattered skeletal element for the grid search method and link search method for the black-back jackal scattering pattern

**Fig. 5:**
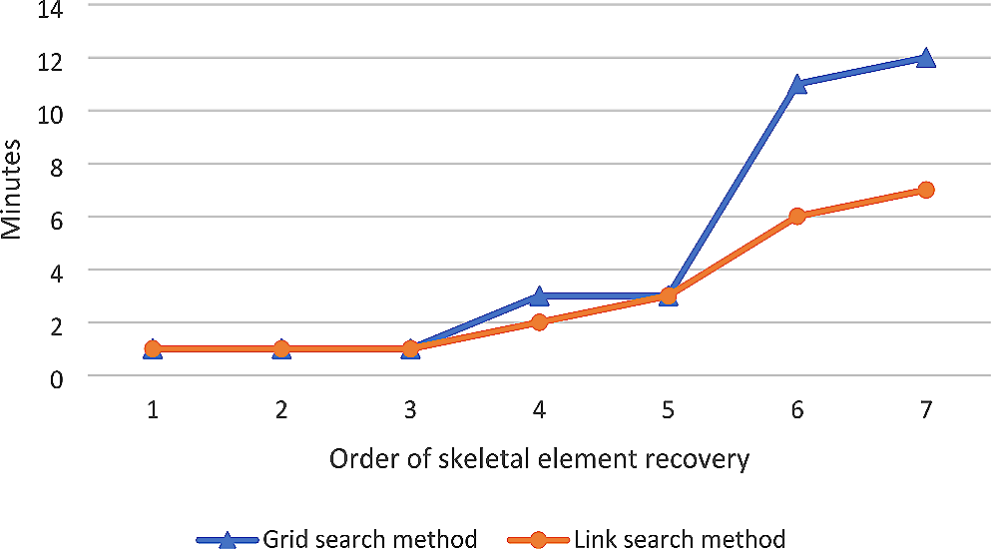
Comparison of the length of time (min) to recover each scattered skeletal element for the grid search method and link search method for the slender mongoose scattering pattern

There was no statistically significant difference in the time taken to recover the scattered remains (jackal scattering pattern: *p* = 0.089; mongoose scattering pattern: *p* = 0.464) between the two search methods. Although there was no statistical difference, there was a difference that was meaningful for practical implementation. The link search method was the fastest method of recovery for both scattering patterns, with a total search time of 26 min for the black-backed jackal scattering pattern (compared to 52 min for the grid method) (Figs. 4) and 12 min or the slender mongoose scattering pattern (compared to 16 min for the grid method) (Fig. 5).

### Participant perceptions of the grid and link search methods

The participant questionnaire answers largely reflected positive perceptions of both the grid and link search methods. The link search method scored higher in perceptions on likeliness of being recommended for use, effectiveness, efficiency, and overall positive experience. It also scored high for perceptions on the usefulness of scavenger behavior knowledge for the location of scattered remains. However, the link search method had more varied perceptions on the ease of its implementation, ease of understanding the method, and perceptions on if it should be developed further (Table 1).

**Table 1:**
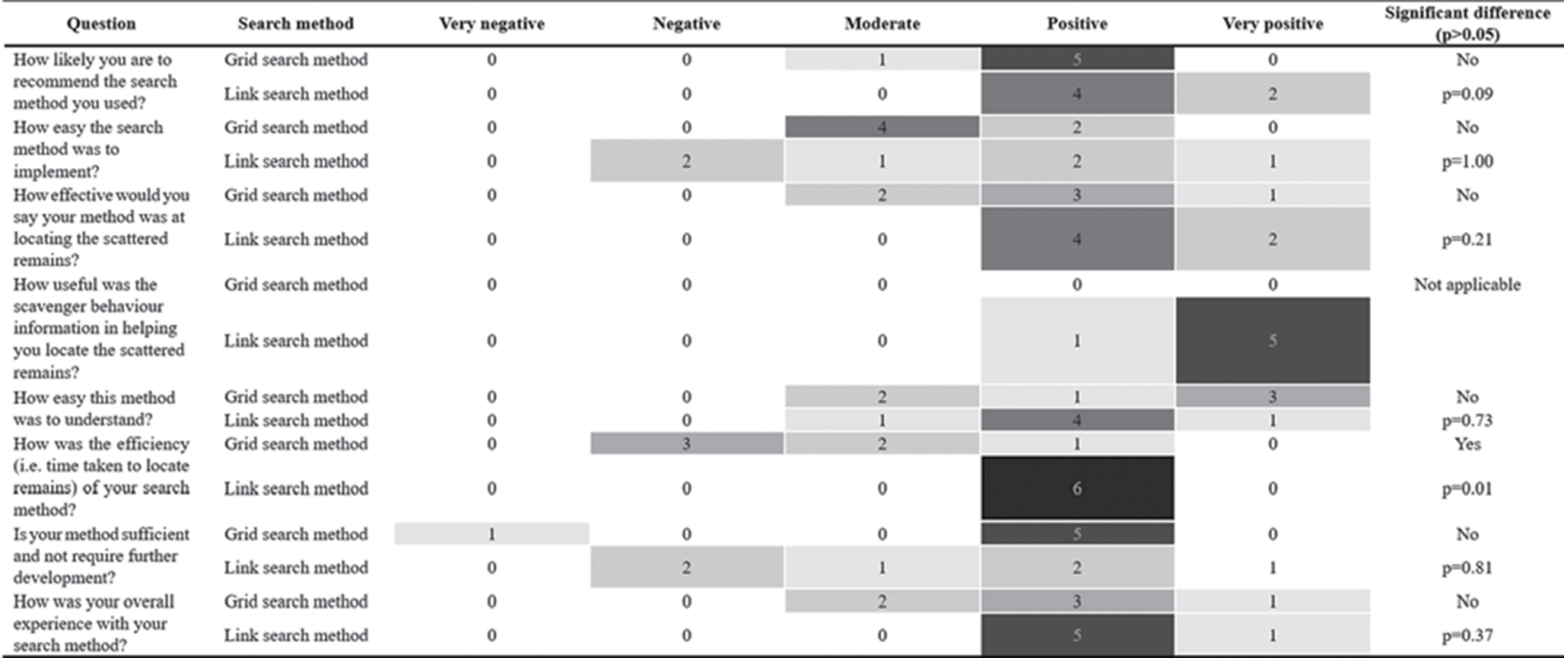
Frequency of answers to a Likert Scale questionnaire on the grid and link search methods

The questionnaire scores for the grid search method indicated mixed perceptions on its effectiveness, ease of use, efficiency, and overall experience. This method scored high for perceptions on if it would be recommended for use and participants indicated that they thought that the method does not require further development (Table 1).

Despite these general patterns indicating slight differences in perceptions between the two search methods, there was no statistically significant difference between the answers for each search method, except for one question. The link search method scored significantly more favorably (*p* = 0.01) for perceptions on the efficiency of the method (i.e. time taken to locate skeletal remains) (Table 1).

## Guidelines for the link search method

There are no descriptive guidelines published on how to perform the link search method. Based on the experience of the author, the feedback from the study participants, and other published literature, the following guidelines are suggested for the implementation of the link search method when recovering remains that have been scattered by scavenging animals.

**Required knowledge**:

1. The search team must have knowledge of which scavenging animals make up the scavenger guild at the scene, especially which species is the dominant scavenger, as their behavior will have the greatest impact on the scattering pattern [11, 12, 14].
  - Species-specific scavenging behaviors impact the scattering direction, scattering range, and skeletal element preference [11, 12, 14].
  - Potential scavengers may be identified by locals in the area or evidence at the site such as paw prints, animal scat, and bone modification patterns [10–12, 15].
2. The search team must have knowledge of how the environment impacts the direction of scattering.
  - Scattering is often towards animal burrows [11].
  - Scattering by small mammalian scavengers is often towards vegetation coverage (such as trees and bushes) [12, 16].
  - Scattering is often along established game trails [11].
  - Scattering rarely extends beyond physical barriers such as fences, walls, or cliffs [11].

**Search guidelines**:

1. It is most effective (but not required) to locate the original site of body deposition and start to search for scattered remains from that site. The original site of body deposition is usually located at the site with the following indicators:
  - The largest concentration of skeletal elements – usually ribs [17, 18].
  - The ground may have numerous pits or signs of foliage disruption due to small mammalians and avians searching for arthropods that migrate away from a body to pupate [12].
2. Determine the possible scattering direction using visual cues [8, 9, 19].
  - Environmental cues include burrows, tree cover, game trails, and physical barriers.
  - Skeletal element cues include the general direction or pattern of skeletal element scatter as observed by the search team.
3. The search team may include a single individual; however, it is recommended that a team of several individuals use a walk-the-line approach (i.e. individuals walk in a line, approximately one meter apart from each other, at a steady pace determined by a team leader) [3].
4. The search team must not follow a rigid straight line, but rather adjust their line search as directed by visual cues.
5. As skeletal elements are located, they should be flagged, recorded, mapped, and photographed and left in situ until the search is complete [5]. It is suggested that the remains be left in situ as the scattering pattern will become apparent as more remains are located and mapped.
6. When the search team reaches a boundary or determines that they have reached or exceeded the scattering range, the search team should return to the deposition or origin site and walk in another direction as prompted by new visual cues.
7. The search will end as determined by the team leader, when no new visual cues are available, the rate of skeletal recovery has stopped, and the known scattering range of the dominant scavenging animal in the area has been reached or exceeded.
8. Remains should only be recovered after all surviving remains have been located, flagged, mapped, and photographed.

## Discussion

Effective search methods are required to ensure the recovery of as many surviving skeletal remains after scavenger scattering as possible. This will ensure a more complete forensic anthropological analysis of skeletal trauma, a more complete biological profile, and adequate forensic case resolution [3, 11, 12, 14]. Both the grid and link search methods proved to be effective, resulting in the recovery of all scattered skeletal remains in both recreated animal scattering patterns. In cases of animal scavenging and scattering of skeletal remains, it is suggested that the link search method be used, especially if the case is time sensitive. The link search method proved to be more efficient because the recovery time was more rapid. Despite there being no statistically significant difference in the length of time to recover the remains between the two methods, the shorter time afforded by the link method makes it more practical for implementation. This is because the link method allows for flexibility and the searcher’s experience to direct the search pattern, unlike the grid method, which relies on strict methodical searching in a rigid grid-like pattern, which is time consuming.

The conditions under which the link search method was tested in this study were very specific: the species guild in the study environment and their scavenging and scattering behaviors were known, the remains were in a state of surface scatter, in subaerial grassland. This method needs further investigation under various conditions, environments, and climates. Several variables have been suggested to impact physical searches for scattered remains, under which each search method should be tested. These variables include environments with different topography, weather, variable periods of subaerial exposure, variable stages of decomposition, skeletal element size, plant cover density, and sunlight dappling caused by tree canopies [3, 9, 20]. The grid search method has previously tested and proven effective in both small and large indoor and outdoor locations, but the link search method has only been tested in outdoor environments [9] and needs further testing in indoor locations, and outdoor locations with varying plant cover densities.

Knowledge of scavenger behaviors and previous experience with scattered human remains and animal proxies have proven to assist searchers to problem solve and adapt their search parameters effectively [9]. This makes the link search method more efficient than the grid search method when the scavenger guild is known, or scavenger behaviors are known to the searchers. However, future studies should also test the efficiency and effectiveness of the link search method where the scavenger guild and their behaviors are unknown to search parties, to assess if specific knowledge of local scavengers is required or if general scavenger behavior knowledge is sufficient.

Participants in the present study indicated in their questionnaire answers that, although they found the link search method to be efficient, it required further development to improve the ease of understanding and implementation of the method. Guidelines have been provided by this study to help address these limitations. Since these are the first formalized and detailed guidelines for the method, it is encouraged that they be further tested and developed to ensure that the guidelines are robust, applicable, and easy to understand and implement.

## Conclusion

In forensic cases where skeletal remains have been scavenged and scattered by animals, it is suggested that the link search method be used for recovery. Knowledge of animal scavenging and scattering behaviors increases the rate of recovery if searchers follow scavenger cues observed in the field. Guidelines for the link search method have been developed in this study to improve the ease of implementation; however, these guidelines need to be tested in different environments to further develop the method and improve its applicability.

## Data Availability

Data sharing is not applicable to this article as no datasets were generated or analyzed during the current study.
